# Protective immunity induced by *Eimeria* common antigen 14–3-3 against *Eimeria tenella, Eimeria acervulina* and *Eimeria maxima*

**DOI:** 10.1186/s12917-018-1665-z

**Published:** 2018-11-12

**Authors:** Jianhua Liu, Lianrui Liu, Lingjuan Li, Di Tian, Wenyu Li, Lixin Xu, Ruofeng Yan, Xiangrui Li, Xiaokai Song

**Affiliations:** 1 MOE Joint International Research Laboratory of Animal Health and Food Safety, College of Veterinary Medicine, Nanjing Agricultural University, Nanjing, 210095 People’s Republic of China; 2 Henan Muxiang Veterinary Pharmaceutical Co., ltd, Zhengzhou, 450000 People’s Republic of China

**Keywords:** Chicken coccidian, Common antigen 14–3-3, Mixed infection, Immunogenicity, DNA vaccine

## Abstract

**Background:**

Avian coccidiosis is often caused by co-infection with several species of *Eimeria* worldwide. Developing a multivalent vaccine with an antigen common to multiple *Eimeria* species is a promising strategy for controlling clinical common co-infection of *Eimeria*. In the previous study, 14–3-3 was identified as one of the immunogenic common antigen in *E. tenella*, *E. acervulina* and *E. maxima*. The aim of the present study was to evaluate the immunogenicity and protective efficacy of Ea14–3-3 in the form of DNA vaccine against infection with three species of *Eimeria* both individually and simultaneously.

**Results:**

After vaccination with pVAX-Ea14–3-3, the Ea14–3-3 gene was transcribed and expressed in the injected muscles. Vaccination with pVAX-Ea14–3-3 significantly increased the proportion of CD4^+^ and CD8^+^ T lymphocytes and produced a strong IgY response in immunized chickens. Similarly, pVAX-Ea14–3-3 stimulated the chicken’s splenocytes to produce high levels of Th1-type (IFN-γ, IL-2) and Th2-type (IL-4) cytokines. The vaccine-induced immune response was responsible to increase weight gain, decreased the oocyst output, and alleviated enteric lesions significantly in immunized chickens as compared to control group, in addition to induce moderate anti-coccidial index (ACI).

**Conclusion:**

These results indicate that Ea14–3-3 is highly immunogenic and capable to induce significant immune responses. Furthermore, Ea14–3-3 antigen can provide effective protection against infection with *Eimeria tenella*, *Eimeria acervulina*, *Eimeria maxima* both individually and in combination with three *Eimeria* species*.* Significant outcomes of our study provide an effective candidate antigen for developing a multivalent *Eimeria* vaccine against mixed infection with various *Eimeria* species under natural conditions.

**Electronic supplementary material:**

The online version of this article (10.1186/s12917-018-1665-z) contains supplementary material, which is available to authorized users.

## Background

Avian coccidiosis is one of the most widespread and economically detrimental diseases in the poultry industry. It causes severe damage to the host intestine, resulting in impaired feed intake, increased mortality and increased susceptibility to other disease agents [1, 2]. It has been estimated that the annual loss due to coccidiosis exceeds $3 billion USD globally [3]. Primarily, control of avian coccidiosis is based on the use of anti-coccidial drugs and live vaccines [1]. Although these two approaches have been generally effective for controlling the disease, the drawbacks of these two approaches, such as drug resistance and residue, high cost and lack of uniformity in vaccination have prompted the search for new generation vaccines including subunit vaccines and DNA vaccines [3–6]. The causes of clinical coccidiosis in the intensive farming are infection by various *Eimeria* species [3, 7]. The species of *Eimeria tenella*, *E. acervulina* and *E. maxima* are commonly found in all commercial birds [1, 8, 9]. Thus, applied vaccines should contain protective antigens common to relevant species and confer effective protection against mixed infection with *Eimeria* species [10].

Several antigens common to *Eimeria* have been reported previously. Talebi et al. [11] found an immunogenic protein (45 kDa) among five *Eimeria* species. While, Sasai et al. [12] observed a common antigen present on conoid of six chicken’s *Eimeria* sporozoites. Additionally, Constantinoiu et al. [13] reported highly conserved apical antigens among subjected *Eimeria* species. However, the reported common antigens were not well identified by sequencing and their protective efficacies have not been evaluated. In the earlier study of our lab, Ea14–3-3 antigen was identified as one of the common immunodominant antigens from *E. tenella*, *E. acervulina* and *E. maxima* [14]. It has been documented that 14–3-3 proteins are involved in many patho-physiological and cellular immune processes by triggering or interfering with the activity of specific protein associates [15]. In apicomplexan parasites, the 14–3-3 protein plays a vital role in parasite invasion, molecular and biological processes with immuno-protective responses [16–20]. Moreover, in *E. tenella*, 14–3-3 antigen was proven to interact with telomerase activity and involved in the process of coccidia development [17].

The 14–3-3 proteins have been showed an immunogenic response, and able to stimulate the host immune activity in some parasites. Schechtman et al. [21] reported that the 14–3-3 antigen of *Schistosoma mansoni* influenced the significant humoral and cellular responses and induced moderate protection against challenged infection. In *Toxoplasma gondii*, the 14–3-3 protein induced effective immune responses in BALB/c mice and was suggested as a novel DNA vaccine candidate against toxoplasmosis [20]. Besides that, 14–3-3 antigens in *Trichinella spiralis* and *Echinococcus* were documented to be highly immunogenic and described as promising vaccine targets against infections [22, 23]. Therefore, 14–3-3 protein may be potential vaccine candidates against these parasites.

In the current study, the immunogenicity and protective efficacy of Ea14–3-3 against *E. tenella*, *E. acervulina* and *E. maxima* was further investigated. Results of this study may provide an effective candidate antigen for developing a multivalent *Eimeria* vaccine against mixed infection with multiple species of *Eimeria* under natural conditions.

## Results

### Sequence analysis and eukaryotic expression plasmid construction of Ea14–3-3 gene

The ORF of Ea14–3-3 gene was cloned into pMD18-T and confirmed by sequencing. The ORF of Ea14–3-3 gene is composed of 837 nucleotides with predicted molecular weights of 31.77 kDa (Additional file 1: Figure S1). Sequence analysis showed that Ea14–3-3 has similarity of 100% in nucleotides and amino acid sequences with the genes in NCBI (XM_013394831). Ea14–3-3 has a high amino acid similarity of more than 94% among the four chicken coccidia species (Additional file 2: Table S1). The constructed pVAX-Ea14–3-3 plasmid was confirmed by endonuclease cleavage and sequence analysis. Endonuclease cleavage with *Bam*H I /*Xho* I produced a band of about 837 bp, which is equal to the size of the inserted gene *Ea14–3-3* (Additional file 3: Figure S2 lane 2). The fragment was extracted from the gel and sequenced. The sequence analysis revealed that the inserted gene has 100% similarity of nucleotides and amino acid sequences with the Ea14–3-3 gene.

### Ea14–3-3 was transcribed and expressed in the injected site of chickens

**Fig. 1:**
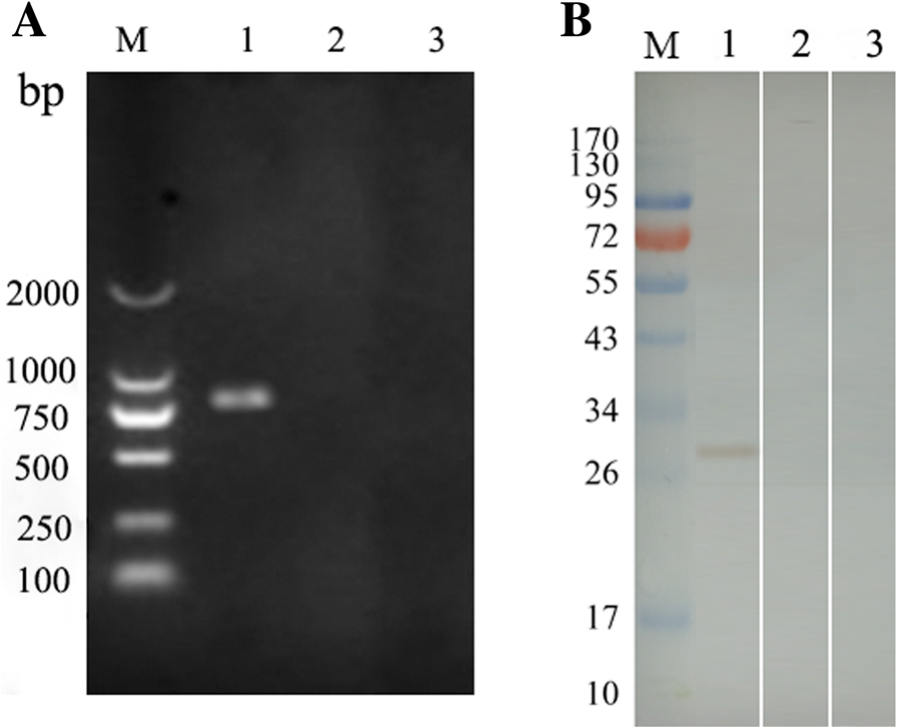
Transcription and expression detection of *Ea14–3-3* gene in injected muscle. **a** RT-PCR analysis of chicken muscles injected with pVAX-Ea14–3-3 M: DL2000 Marker. Lane 1: RT-PCR product of chicken muscles injected with pVAX-Ea14–3-3. Lane 2: non-injected muscle. Lane 3: pVAX1 plasmid injected muscle. **b** Western blot analysis of chicken muscles injected with pVax-Ea14–3-3; M: Protein molecular weight marker. Lane 1: Western blot analysis of chicken muscles injected with pVAV-Ea14–3-3. Lane 2: non-injected muscle. Lane 3: pVAX1 plasmid injected muscle

RT-PCR assay was employed with the specific primers for Ea14–3-3 to detect transcriptions of the Ea14–3-3 gene in the injected muscles. Agarose electrophoresis showed a band of approximately 837 bp from the muscle injected with pVAX-Ea14–3-3 (Fig. 1a, lane 1). No specific DNA bands were detected in pVAX1 injected and non-injected control sample (Fig. 1a, lane 2 and 3). These results indicate that Ea14–3-3 was transcribed in the injected site muscles of chickens.

Western blot assay was employed with primary antibody of anti-*E. acervulina* chicken sera to detect the expressed proteins. As shown in Fig. 1b, in pVAX-Ea14–3-3 injected muscle anti-*E. acervulina* chicken sera reacted with a protein band of approximately 32 kDa (Fig. 1b, lane 1). No specific band was detected in non-injected control and vector control samples (Fig. 1b, lane 2 and 3). These results indicate that Ea14–3-3 was expressed in the injected site muscles of chickens.

### Ea14–3-3 induced significant cellular immune responses in chickens

**Fig. 2:**
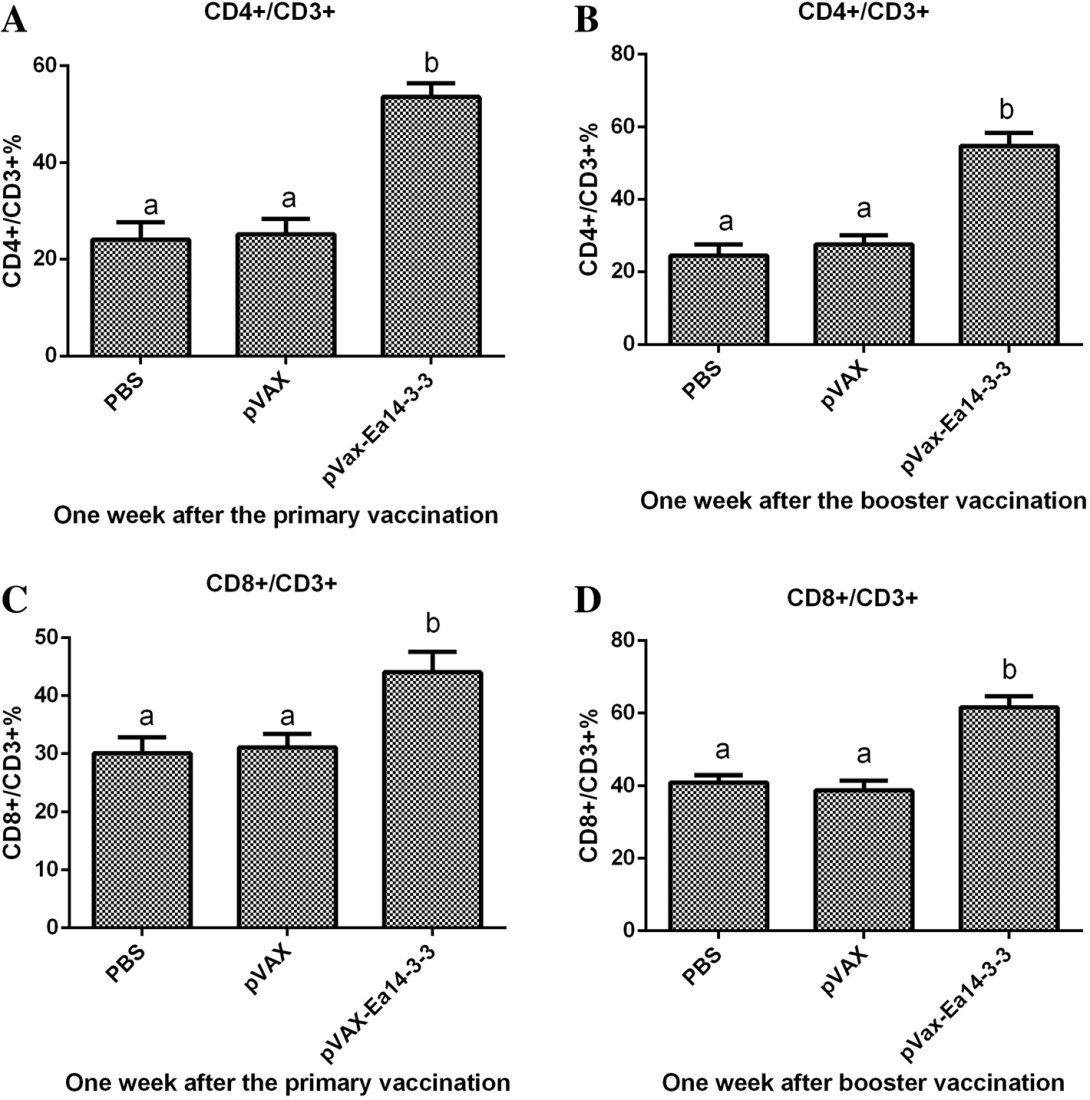
Changes of proportion of CD4^+^/CD3^+^ and CD8^+^/CD3^+^ T cells in spleens of the chickens vaccinated with pVAX-Ea14–3-3. (**a**) Proportion of CD4^+^/CD3^+^ T cells in spleens of the chickens 1 week after the primary vaccination; (**b**) proportion of CD4^+^/CD3^+^ T cells in spleens of the chickens 1 week after the booster vaccination; (**c**) proportion of CD8^+^/CD3^+^ T cells in spleens of the chickens 1 week after the primary vaccination; (**d**) proportion of CD8^+^/CD3^+^ T cells in spleens of the chickens 1 week after the booster vaccination; significant difference (*p* < 0.05) between numbers with different letters; non-significant difference (*p* > 0.05) between numbers with the same letter

**Fig. 3:**
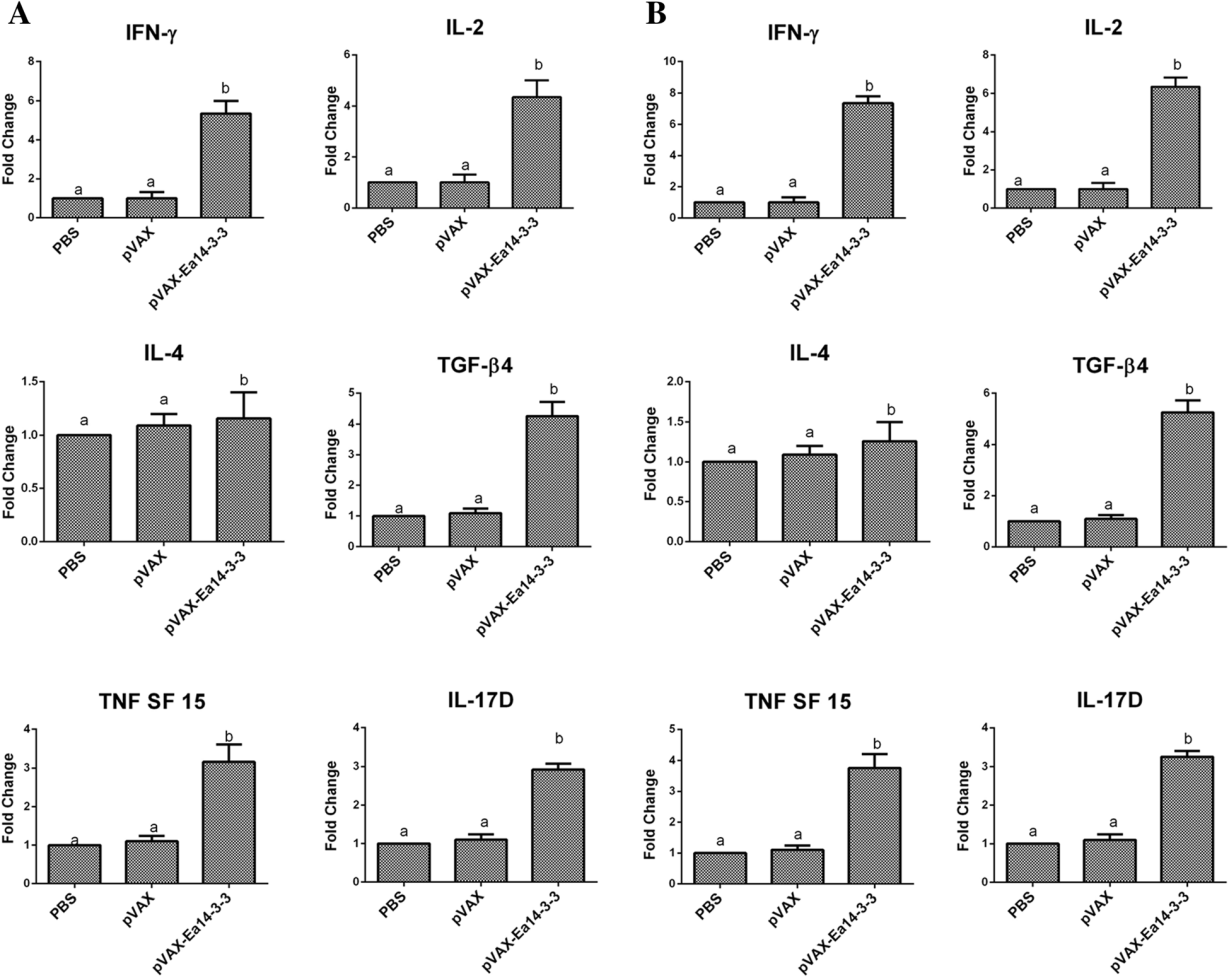
Changes of mRNA expression of cytokines in splenic lymphocytes following pVAX-Ea14–3-3 vaccination. **a** One week after the primary vaccination; (**b**) One week after the booster vaccination; Significant difference (*p* < 0.05) between numbers with different letters; non-significant difference (*p* > 0.05) between numbers with the same letter

The proportions of CD4^+^/CD3^+^ and CD8^+^/CD3^+^ splenic T lymphocytes from vaccinated chickens were determined by flow cytometry assay. As per depicted in Fig. 2, 1 week after the primary and booster dose of vaccination, the proportions of CD4^+^/CD3^+^ and CD8^+^/CD3^+^ splenic T lymphocytes from vaccinated chickens were significantly higher than those from PBS and pVAX1 empty plasmid control chickens (*p* < 0.05). No significant difference was observed between the pVAX1 and PBS control chickens (*p* > 0.05). The results demonstrated that Ea14–3-3 effectively promoted the T lymphocyte responses in chickens. The mRNA levels of IFN-γ, IL-2, IL-4, TNFSF15, IL-17D and TGF-β4 cytokines from vaccinated chickens were determined by qPCR. As shown in Fig. 3, 1 week after the primary and booster dose of immunization, the production of IFN-γ, IL-2, IL-4 TNFSF15, IL-17D and TGF-β4 was significantly increased in pVAX-Ea14–3-3-vaccinated chickens as compared to pVAX1-vaccinated and PBS control chickens *(p* < 0.05). No significant difference was observed between pVAX1 and PBS control chickens (*p* > 0.05). These results indicated that Ea14–3-3 effectively promoted the production of cytokines in chickens.

In a word, the *Eimeria* common antigen 14–3-3 effectively induced the secretion of cytokines in chickens.

### Ea14–3-3 induced a significant serum antibody response in chickens

**Fig. 4:**
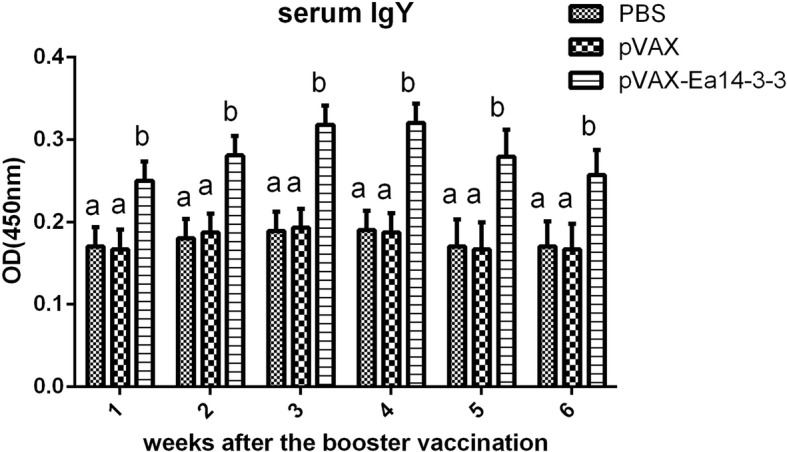
Serum specific IgY levels in chickens following the recombinant plasmid pVAX-Ea-14-3-3 vaccination. At weeks 1, 2, 3, 4, 5 and 6 post-second immunization, blood was collected by cardiac puncture and antibody levels were determined by ELISA. Each bar represents the mean ± S.D. (*N* = 5). Values with different superscripts in the same differ significantly (*P* < 0.05)

The indirect ELISA method was employed to determine the antibody response induced by Ea14–3-3*.* As shown in Fig. 4, from 1 week to 6 weeks post-booster vaccination, the induced antibody titers of all pVAX-Ea14–3-3 vaccinated groups were significantly higher as compared to the PBS and pVAX1 empty plasmid control groups. No significant difference was observed between the pVAX1 and PBS control group (*p* > 0.05). The antibody titers of vaccinated groups increased slowly from the first week to the third week post-booster immunization, peaked at the fourth week, and then decreased gradually PBS and pVAX1 empty plasmid groups were not detected any specific antibodies. The results showed that Ea14–3-3 significantly induced significant serum antibody response in chickens.

### Ea14–3-3 induced effective protection against *E. tenella*, *E. acervulina* and *E. maxima*

To evaluate the protective efficacy of pVAX-Ea14–3-3, groups of chickens were challenged with *E. tenella*, *E. acervulina*, *E. maxima* or mixed oocysts of the three *Eimeria* species. As shown in Table 1, vaccination with pVAX-Ea14–3-3 significantly increased the body weight gains, decreased the oocyst output, and alleviated the enteric lesions of vaccinated groups as compared to PBS control and pVAX1 control groups (*p* < 0.05). No significant difference was observed between the pVAX1 and PBS control group (*p* > 0.05). The vaccination resulted in ACIs of 171.31(*E. acervulina*), 161.03(*E. maxima*), 178.29 (*E. tenella*) and 170.92 (mixed *Eimeria*). This result indicates that Ea14–3-3 provided effective protections against challenge with *E. tenella, E. acervulina, E. maxima* and mixed infection of the three *Eimeria* species.

## Discussion

Avian coccidiosis, one of the most potential destructive diseases in birds, is caused by several *Eimeria* species under natural conditions. Thus an ideal field *Eimeria* vaccine should provide effective protection against co-infection with mixed *Eimeria* species [2, 9, 24–26]. Common antigens shared among *Eimeria* species are extremely promising for the development of multivalent *Eimeria* vaccines against multiple *Eimeria* species. Previous studies have revealed such a few common antigens among *Eimeria* which had serological cross reactions among certain species [13]. For example, Talebi [11] found a few shared proteins between species and at least one protein band (45 kDa) was conserved among the five species. The conserved protein band of all these species could be recognized by chicken anti-*E. maxima* sera. Sasai et al. [12] identified a common conoid sporozoites antigen among 6 different *Eimeria* species (*E. brunetti*, *E. maxima*, *E. mitis*, *E. necatrix*, *E. praecox* and *E. tenella*) by confocal laser scanning microscopy using a chicken monoclonal antibody (mAb) 6D12-G10 against *E. acervulina* sporozoite. Constantinoiu et al. [13] analyzed cross-reactivity of five chicken mAb against *E. acervulina* sporozoites using confocal laser immunofluorescence assay and found mAb 8E-1 recognized an apical tip molecule present on all chicken *Eimeria* sporozoites. However, these above studies merely revealed molecular weight or fluorescence localization of the common antigens. They did not further identify the specific common antigen by individual sequencing. In our previous study, at least 5 specific *Eimeria* common immunodominant antigens among three *Eimeria* species have been identified by immuno-proteomic analysis and LC-MS/MS technique [13]. The 14–3-3, one of the five identified *Eimeria* common immunogenic antigens is highly conserved among three *Eimeria* species (Additional file 2: Table S1). In the present observation, we further evaluated immunogenicity and protective efficacy of common antigen of 14–3-3 against challenge with *E. tenella*, *E. acervulina* and *E. maxima*. We found that vaccination with the common antigen Ea14–3-3 contributed effective protection not only against individual infection with *E. tenella, E. acervulina,* or *E. maxima*, but also against the co-infections with corresponding species. Hence, these findings provide a promising common antigen for developing a vaccine against clinical co-infection by multiple *Eimeria* species.

Cellular immune responses play a dominant role in the immunity against coccidiosis [27]. In this study, the cellular immune responses induced by *Ea-*14-3-3 were assessed. The results showed that the proportions of spleen T lymphocyte subpopulations of CD4^+^ and CD8^+^ were significantly increased. High levels of Th1-type (IFN-γ, IL-2) and Th2-type (IL-4) cytokines were produced. These vaccine-induced immune responses resulted in effective protections against *Eimeria*. These results are consistent with other reported findings. Bessay et al. [28] found that *E. acervulina* infection induced a significant increase in the proportion of CD4^+^ and CD8^+^ in the duodenal intraepithelial leucocytes (IEL) from day 4 to day 8 post infection (pi). Min et al. [29] found that the ratios of CD4^+^/CD3^+^ and CD8^+^/CD3^+^ were remarkably increased after immunizing chickens with a pcDNA3-1E vaccine.

Several cytokines have been shown to be involved in immune responses to *Eimeria* infection [27, 30]. Th1-type cytokines such as IFN-γ and IL-2 are responsible for cellular immunity and dominant during *Eimeria* infection [27, 30]. The cytokine IFN-γ has been demonstrated to be important in immuno-regulation in coccidial infections [26, 31]. Recombinant chicken IFN-γ could inhibit the intracellular development of *E. tenella* in vitro and reduce oocyst production and body weight loss following *E. acervulina* challenge infection [30, 32]. IL-2 is considered as a potent growth factor for T-cell differentiation, B-cell development and NK-cell activation [30, 33]. IL-2 mRNA transcripts level in the spleen and intestine was significantly enhanced after infections with *E. acervulina*. IL-2 has been demonstrated to be able to significantly improve the protective effect of recombinant coccidia genes by DNA vaccination [29, 34]. In this study, the mRNA transcripts level of IFN-γ and IL-2 were significantly increased by vaccination with pVAX-Ea14–3-3 (Fig. 3). Furthermore, proportions of CD4^+^/CD3^+^ and CD8^+^/CD3^+^ T lymphocytes were significantly increased (Fig. 2). The increasing trend of CD4^+^/CD3^+^ and CD8^+^/CD3^+^ T lymphocytes in accordance with IFN-γ and IL-2, indicating that T-cell immune response might be prompted by Th1-type cytokines. IL-4, a typical Th2-type cytokine, is responsible for regulating humoral immunity [35]. In this study, the mRNA transcripts level of IL-4 was significantly increased by vaccination with pVAX-Ea14–3-3 (Fig. 3), which in accordance with high level of antibody response in the vaccinated chickens. IL-17D produced by Th17 cells, participates in the induction of inflammation during protozoan infection. After infection with *E. maxima* and *E. tenella*, the expression of IL-17 increased significantly in lymphocytes of the spleen [36, 37]. In this study, the mRNA transcripts level of IL-17D was significantly increased by vaccination with pVAX-Ea14–3-3 (Fig. 3). TGF-β is an anti-inflammatory cytokine that downregulate inflammatory responses and promote repair of damaged mucosal epithelial integrity following injury [30, 38]. After infected with *E. acervulina*, the mRNA level of TGF-β4 in spleen and intestinal epithelial endothelial cells was significantly higher than that in uninfected chickens [39]. In this study, the mRNA level of TGF-β4 was significantly increased by vaccination with pVAX-Ea14–3-3. The high level of TGF-β4 might help repair the mucous membrane damaged by *Eimeria* parasites. TNF is able to promote the proliferation and differentiation of IL-2 and IFN-γ, enhancing the stimulation of antigen on B cells [40]. In vivo experiments revealed that TNFSF15 gene was highly increased following primary infections with *Eimeria* [41]. In this study, the mRNA level of TNFSF15 was significantly increased following vaccination with pVAX-Ea14–3-3. In short, vaccination with pVAX-Ea14–3-3 induced high level of IFN-γ, IL-2, IL-4, TNFSF15, IL17D and TGF-β, playing important roles in immune responses against *Eimeria* infection.

Although the Ea14–3-3 DNA vaccine provided effective protection against *Eimeria*, some measures could be taken to improve protection and to make it more practical for use in fields [42]. The protective efficacy of the DNA vaccine could be enhanced by co-injection with plasmids encoding immune stimulating cytokines (IFN-γ, IL-2) [29, 34, 43]. The sugarcane (*Saccharum officinarum* L.) bagasse-derived polysaccharides could be used as native immunomodulatory candidate to improve the protective efficacy of the DNA vaccine [44].

In the current study, an optimal vaccination procedure including two injections was used to obtain best efficacy for the DNA vaccine. However, on commercial broiler poultry farms, farmers prefer to administer single vaccinations via non-injection delivery routes at day 1 of age. Hence, the vaccination procedure must be further optimized to make vaccination with pVAX-Ea14–3-3 more practical, for example, identify the minimal vaccination age and evaluate the efficacy of a single vaccination and non-injection delivery routes [45].

## Conclusion

The coccidal common antigen of Ea14–3-3 induced significant humoral and cellular immune response against *Eimeria* infection. Vaccinations with DNA vaccine of Ea14–3-3 had significant ability to induce effective protection against infection of individual *Eimeria* species (*E. tenella, E. acervulina* and *E. maxima*), while, also in the mixed infection of these species. Our study indicates that effective common antigen of 14–3-3 could be used in the development of multivalent vaccine against co-infections of multiple *Eimeria* species in commercial poultry industry.

## Methods

### Chicken, parasite and vector

One-day-old Hy-Line layer chickens were conventionally reared in standardized and sterilized wire cages to prevent intensive contact with any contamination. The birds were given with coccidiostat-free feed and water ad-libitum. Animal experiment was approved by the Institutional Animal Care and Use Committee of Nanjing Agricultural University (approval number: 2012CB120762). Oocysts of *E. tenella*, *E. acervulina* and *E. maxima* used in challenged infection were propagated, harvested and sporulated 7 days prior to the challenged infection, using a previously described protocol [46]. The eukaryotic expression vector pVAX1 was purchased from Invitrogen (Carlsbad, California, USA).

### Antisera preparation

Two-week-old chickens were orally inoculated with 1 × 10^4^ sporulated oocysts of *E. acervulina* 3–5 times per bird by the interval of 3-days. Negative control birds were inoculated with distilled water. One week post the third inoculation, wing vein blood was collected and determined by ELISA.Forth to fifth dose was given to chickens unless titers of the sera antibody were beyond 1: 64. Serum was stored at − 20 °C for Western blot analysis, while negative control serum was collected from negative control chickens.

### Cloning of the *14–3-3* gene

Micro glass beads were used to break the *E. acervulina* sporulated oocysts via whirl mix [46]. Briefly, equal volume of 0.5-mm-diameter glass beads and oocysts were mixed in a tube and agitated on a whirl mix with the maximum speed for about 8–10 bursts of 1 s each. At the interval of every 4 bursts, supernatant sample was scrutinized by microscopic examination to ensure that most of the oocysts were broken and sporocysts were intact. Subsequently, the sporocysts were recovered from the glass beads by repeated additions of medium. The sporozoites were released from the sporocysts by in vitro excystation with trypsin 0.25% (*w*/*v*) and taurocholic acid 1% (w/v) at 41 °C. Finally, the sporozoites were purified over nylon wool and DE-52 cellulose columns according to the manufacturer’s instructions [46]. Total RNA was extracted from *E. acervulina* sporozoites using E.Z.N.A. TM Total RNA Kit I (OMEGA, Norcross, Georgia, USA) according to the manufacturer’s instructions. Reverse transcription reaction (RT) was performed to produce cDNA with Oligo (dT) as primers [47]. With the complementary DNA (cDNA) as a template, the complete open reading frame (ORF) of the Ea14–3-3 gene (GenBank Accession No. XM_013394831) was amplified by polymerase chain reaction (PCR) using specific primers designed for Ea14–3-3 ORF (*BamH* I anchored forward primer 5’-CGCGGATCCATGATTGAGGACATCAAGACTCTT-3′, *Xho* I anchored reverse primer 5’-CCGCTCGAGCTACTGCTGCTCAGTAGTAGCTT-3′). The PCR products were cloned into pMD18-T vector (TaKaRa Biotech, Dalian, China) to produce pMD18-T-Ea14–3-3. The resultant plasmid was identified by endonuclease digestion and sequencing. Basic local alignment search tool (BLAST) (http://www.ncbi.nlm.nih.gov/BLAST/) was used to analyze the nucleotide sequence.

### Construction of eukaryotic expression plasmid pVAX-Ea14–3-3

The ORF of *Ea14–3-3* was cloned into eukaryotic expression vector pVAX1 to construct pVAX-Ea14–3-3. Briefly, fragments of Ea14–3-3 were excised from the pMD18-T-Ea14–3-3 by *BamH* I and *Xho* I digestion and ligated into pVAX1 at the same enzyme sites to construct pVAX-Ea14–3-3. The resulting plasmid was confirmed by endonuclease cleavage and sequence analysis.

### Transcription detection of the Ea14–3-3 gene by RT-PCR in chickens

Two-week-old chickens were vaccinated with 100 μg dose of the recombinant plasmid pVAX-Ea14–3-3 through intramuscular injection in the thigh region. While, control group chickens were injected with the pVAX1 vector as described above. A small circle was marked on the injection site and kept clear until the cutting of muscle sample. One week later, injected site muscle sample (~ 0.5 g) was excised from each chicken for mRNA extraction. Potential residual plasmids were removed by DNase I (TaKaRa) digestion. RT-PCR was employed with the RNA product as template using the specific primers for Ea14–3-3 ORF. Electrophoresis in 1% agarose gel was subsequently performed to detect the Ea14–3-3 fragment*.* Muscles from the corresponding site of non-injected chickens were also lacerated as control samples.

### Expression detection of Ea14–3-3 gene by Western blot in chickens

Two-week-old chickens were immunized with 100 μg dose of the recombinant plasmid pVAX-Ea14–3-3 or pVAX1 vector as mentioned previously. One week later, a sample of each injected muscles (about 0.5 g) was obtained as before, and treated with RIPA lysis buffer (0.1 mol/L phenylmethylsulfonyl fluoride (PMSF), 50 mmol/L Tris–HCl, 150 mmol/L NaCl, 1% Nonnidet P-40, 0.1% SDS) for 3 h. The muscle sample from the corresponding site of each non-injected chicken was used as the control. The samples were centrifuged at 13,000 rpm for 10 min, and the supernatant was collected for Western blot analysis. For detection of expressed proteins, Western blot assay was performed using anti-*E. acervulina* chicken sera as primary antibody as previously reported method [48, 49].

### Immunogenicity evaluation of Ea14–3-3 in chickens

#### Experimental design

Two-week-old chickens were randomly divided into 3 groups of 40 chickens in each group. As described previously, experimental groups of chickens were vaccinated with 100 μg of recombinant plasmid pVAX-Ea14–3-3. Vector control group chickens were injected with 100 μg of empty pVAX1, and the PBS control group chickens were injected with same volume of sterile PBS (pH 7.4). One week later, all chickens were received booster injection. One week after the primary and booster immunizations, 5 chickens from each group were euthanized by cervical dislocation for evaluation of T lymphocyte sub-populations and cytokines production separately. Blood sera were collected from the rest of 30 chickens in each group for specific antibody determination.

### Flow Cytometry analysis of T lymphocyte subpopulations

Ea14–3-3 antigen induced changes in T lymphocyte subpopulations were determined using flow cytometry. Spleens from 5 euthanized chickens of each group were collected to evaluate spleen’s T lymphocyte subpopulation proportions of CD4^+^ and CD8^+^. The spleens were cut into pieces and gently pushed through a mesh (250 μm pore size). Spleen lymphocyte suspensions were prepared as described previously [50]. The cells (1 × 10^6^ cells/ml) were dually stained with mouse anti-chicken CD3-PE/Cy5, mouse anti-chicken CD8α-FITC, and mouse anti-chicken CD3-PE/Cy5 + mouse anti-chicken CD4-FITC at room temperature in the dark for 30 min. After 3 washes twice PBS by centrifugation (2000 rpm for 5 min at 4 °C), splenocytes population were determined by FACScan flow cytometer and analyzed with Cell Quest software (BD Biosciences, Franklin Lakes, NJ, USA).

### Determination of cytokine transcription by quantitative real-time PCR

Spleen lymphocytes from the vaccinated chickens (five per group) were prepared as previously described [50]. Total RNA was extracted from spleen lymphocytes using an E.Z.N.A.® Total RNA Kit Maxi Kit (OMEGA). The cDNA was then generated using RT-PCR. Quantitative real-time PCR (qPCR) was employed to determine IFN-γ, IL-2, IL-4 TNFSF15, IL-17D and TGF-β4 mRNA levels in immunized chickens. The qPCR was carried out with an initial denaturation at 95 °C for 30 s, followed by 40 cycles at 95°C for 10 s, at 60 °C for 30 s and followed by a melting curve program at 95°Cfor 15 s, at 60°Cfor 15 s, at 95 °Cfor 15 s, using an ABI PRISM 7500 Fast Real-Time PCR System (Applied Biosystems, Carlsbad, CA,United States). The chicken GAPDH gene was used as an internal control. The primers for qPCR are shown in Table 2 [51]. The same cDNA sample (without dilution) was used for all cytokines and GAPDH to normalize and standardize the data. The relative quantification of cytokine gene mRNA was determined via comparison the internal control gene of GAPDH using the 2^-ΔΔCT^ method as previously described [52]. A validation experiment was performed by running a dilution series of the cDNA to evaluate the amplification efficiencies of the cytokine genes and internal control gene [51]. The qPCR efficiencies (E) were calculated using the following formula: E = 10^–1/slope^ − 1 [53]. Pfaffl correction was conducted for these qPCR analyses.

**Table 1:** Protective efficacy of common antigen *Ea14–3-3* against challenge with *E. tenella*, *E. acervulina*, *E. maxima* and mixed oocysts of the three *Eimeria* species

Group	Challenge with *Eimeria* spp.	Average body weight gain (g)	Relative body weight gain (%)	Mean lesion scores	Oocyst decrease ratio (%)	ACI
pVAX-Ea14–3-3	*E. tenella*	53.26 ± 11.52^b^	87.49	0.42 ± 0.52^b^	58.79	178.29
pVAX1 control	*E. tenella*	35.26 ± 9.89^a^	58.00	3.59 ± 0.51^c^	−0.61	112.1
Challenged control	*E. tenella*	32.58 ± 10.29^a^	53.11	3.78 ± 0.67^c^	0.00	105.41
Unchallenged control*	PBS	61.30 ± 9.29^c^	100	0 ± 0^a^	100	200
pVAX-Ea14–3-3	*E. acervulina*	53.96 ± 12.0 ^b^	88.51	1.62 ± 0.54^b^	77.05	171.31
pVAX1 control	*E. acervulina*	38.41 ± 10.93^a^	63.20	3.01 ± 0.81^c^	12.67	93.1
Challenged control	*E. acervulina*	37.38 ± 10.27^a^	62.37	3.26 ± 0.67^c^	0.00	89.77
Unchallenged control*	PBS	61.30 ± 8.89^c^	100	0 ± 0^a^	100	200
pVAX-Ea14–3-3	*E. maxima*	52.96 ± 11.91^b^	88.23	1.72 ± 0.64^bc^	72.04	161.03
pVAX1 control	*E. maxima*	35.39 ± 11.52^a^	58.62	2.56 ± 0.71^c^	12.19	93.02
Challenged control	*E. maxima*	35.18 ± 12.21^a^	57.18	2.90 ± 0.67^c^	0.00	88.18
Unchallenged control*	PBS	61.30 ± 9.29^c^	100	0 ± 0^a^	100	200
pVAX-Ea14–3-3	Mixed oocysts	52.98 ± 11.54^b^	86.82	0.59 ± 0.51^b^	39.33	170.92
pVAX1 control	Mixed oocysts	35.10 ± 10.81^a^	57.57	3.58 ± 0.61^c^	−1.69	111.77
Challenged control	Mixed oocysts	33.08 ± 11.09^a^	53.77	3.70 ± 0.57^c^	0.00	106.77
Unchallenged control*	PBS	61.30 ± 9.29^c^	100	0 ± 0^a^	100	200

Significant difference (*p* < 0.05) between numbers with different letters. No significant difference (*p* > 0.05) between numbers with the same letter

*unchallenged control was shared among the groups challenged with different *Eimeria* species

Mixed oocysts: mixed oocysts of *E. tenella*, *E. acervulina* and *E. maxima*

**Table 2:** Primers used for the quantitative RT-PCR

RNA target	Primer sequence	Accession NO.	Amplification efficiency (%)^a^	Correlation coefficients (r^2^)
IFN-γ	Forward: 5′-AGCTGACGGTGGACCTATTATT-3′	Y07922	99.16	0.9976
	Reverse: 5′-GGCTTTGCGCTGGATTC-3′			
IL-2	Forward: 5′-TCTGGGACCACTGTATGCTCT-3′	AF000631	98.53	0.9932
	Reverse: 5′-ACACCAGTGGGAAACAGTATCA-3’			
TNFSF15	Forward:5′-CCTGAGTTATTCCAGCAACGCA-3′	NM_001024578	98.51	0.9992
	Reverse: 5′-ATCCACCAGCTTGATGTCACTAAC-3′			
IL-17D	Forward:5′-GCTGCCTCATGGGGATCTTTGGTG-3′	EF570583	98.18	0.9954
	Reverse: 5′-CGATGACGGCTTGTTCTGGTTGAC-3′			
TGF-β4	Forward: 5′-CGGGACGGATGAGAAGAAC-3′	M31160	97.39	0.9981
	Reverse: 5′-CGGCCCACGTAGTAAATGAT-3′			
IL-4	Forward: 5′-ACCCAGGGCATCCAGAAG-3′	AJ621735	99.41	0.9996
	Reverse: 5′-CAGTGCCGGCAAGAAGTT-3′			
GAPDH	Forward: 5′-GGTGGTGCTAAGCGTGTTAT-3′	K01458	95.48	0.9994
	Reverse: 5′-ACCTCTGTCATCTCTCCACA-3′			

^a^Amplification efficiency (%) = (10^–1/slope^ − 1) × 100

### Determination of serum antibody level

Blood samples were collected from the wing vein of each chicken at 1-week intervals for 6-weeks post-booster vaccination. Sera were collected for determining Ea14–3-3-specific antibody levels through indirect ELISA as previously described [49]. In brief, 96-well microtiter plates (Corning-Costar NY, USA) were coated overnight at 4 °C with 10 μg/ml *E. acervulina* sporozoites (100 μl protein solution per well) in 0.05 M carbonate buffer (pH 9.6). The plates were washed 3 times with PBST, blocked with 5% Bovine Serum Albumin (BSA) for 2 h at 37 °C and then incubated with chicken serum diluted 1:50 in PBS for 1 h at 37 °C. A 1: 3000 dilution of horseradish peroxidase-conjugated donkey anti-chicken IgY anti-body (Sigma) in 5% SMP was added as the secondary antibody (100 ml/well) to detect bound antibodies, and the plates were again incubated for 1 h at 37 °C. Finally, the complexes were developed by incubation with 3, 3, 5, 5-tetramethylbenzidine (TMB) for 15 min. The reaction was stopped by adding 50 μL of 2 M H_2_SO_4_ to each well, and the absorbance was measured at 450 nm (OD_450_) using an automated ELISA reader.

### Evaluation of immune protection

At 14 days of age, chickens were weighed and randomly divided into 13 groups with 30 chickens per group. Experimental group chickens were immunized with 100 μg of pVAX-Ea14–3-3 via intramuscular injection in thigh region. The challenged control group and unchallenged control chickens were injected with sterile PBS. The empty vector control group was immunized with 100 μg pVAX1 as mentioned above. A booster immunization was given at 7 day after the first immunization. At 28 days of age, the chickens were challenged with freshly sporulated oocyst of *E. tenella* (5 × 10^4^/chicken), *E. acervulina* (1 × 10^5^/chicken) and *E. maxima* (1× 10^5^/chicken) and mixed sporulated oocysts (5 × 10^4^ *E. tenella* /chicken, 1 × 10^5^ *E. acervulina* /chicken, 1 × 10^5^ *E. maxima* /chicken) separately except the non-immunized and non-challenged group [54]. Six days post-challenged infection, all the chickens were slaughtered. Average body weight gain, oocyst decrease ratio, lesion score, and anti-coccidial index (ACI) were calculated.

The protective efficacy was evaluated based on body weight gain, lesion score, oocyst output, oocyst decrease ratio and ACI [55, 56]. Body weight gain was determined by weighing the chickens at the end of the experiment and deducing the weight of the same chickens at the time of challenge. Lesion scores were observed and recorded consistent with the method described by Reid and Johnson [57]. The intestinal contents from the whole guts of chickens in all groups were collected and oocysts per gram of content (OPG) were determined via McMaster’s counting technique. ACI is a comprehensive index for assessing the protective effect of immune protection and is calculated as follow: (survival rate + relative rate of weight gain) - (lesion value + oocyst value). According to McManus [58], an ACI ≥180 is considered as high performance, an ACI between 160 and 179 is considered effective, while value of ACI < 160 is considered ineffective. All the chickens in this study were euthanized by CO_2_ inhalation. Briefly, CO_2_ was delivered from compressed gas canister with flowmeter and pressure regulator. After opening the switch, CO_2_ would be steadily flowing into the euthanasia chamber. The number of animals put in the euthanasia chamber depended on the size of the box to avoid crowding. The entire body of the animal must go into the euthanasia chamber. The gas was delivered with a displacement rate of 20% of the euthanasia chamber volume per minute. After the animal loses consciousness, a secondary physical method of euthanasia of cervical dislocation was performed.

### Statistical analysis

Non-normally distributed ANOVA with Tamhane’s T2 multiple range tests were applied for the determination of statistical significance through SPSS statistical package (SPSS Inc., Chicago, IL, USA). The differences between all groups were tested and *p* < 0.05 value was considered as to indicate a significant difference.

## Additional files

Additional file 1: **Figure S1.** Open reading frames (ORFs) and deduced amino acid sequence of common antigen 14–3-3. (TIF 12897 kb)

Additional file 2: **Table S1.** Amino acid similarities of 14–3-3 between *E. acervulina*, *E. maxima*, *E. tenella*, *E. necatrix* (%). 1.Ea14–3-3 = 14–3-3 of *E. acervulina*; Em14–3-3 = 14–3-3 of *E. maxima*; Et14–3-3 = 14–3-3 of *E. tenella*; En14–3-3 = 14–3-3 of *E. necatrix. (DOCX 16 kb)*

Additional file 3: **Figure S2.** Identification of recombinant plasmid pVAX-Ea14–3-3 digested by *BamH I/Xho I.* M: DNA molecular weight marker DL 2000. Lane 1: pVAX-Ea14–3-3. Lane 2: pVAX-Ea14–3-3 digested by *BamH I/Xho I. (TIF 62 kb)*

## Abbreviations

ACI: Anti-coccidial index
*E. acervulina*: *Eimeria acervulina*
*E. maxima*: *Eimeria maxima*
*E. tenella*: *Eimeria tenella*
Ea14–3-3: *Eimeria acervulina* 14–3-3 protein
FITC: Fluorescein isothiocyanate
ORF: Open reading frame
PE: P-phycoerythrin
qPCR: quantitative real-time PCR
RT-PCR: transcription-polymerase chain reaction
