# Supplementary figures and images for: Protective immunity induced by Eimeria common antigen 14–3-3 against Eimeria tenella, Eimeria acervulina and Eimeria maxima
Source: BMC Vet Res. 2018 Nov 12;14:337. doi: 10.1186/s12917-018-1665-z (PMC6233286; doi:10.1186/s12917-018-1665-z)

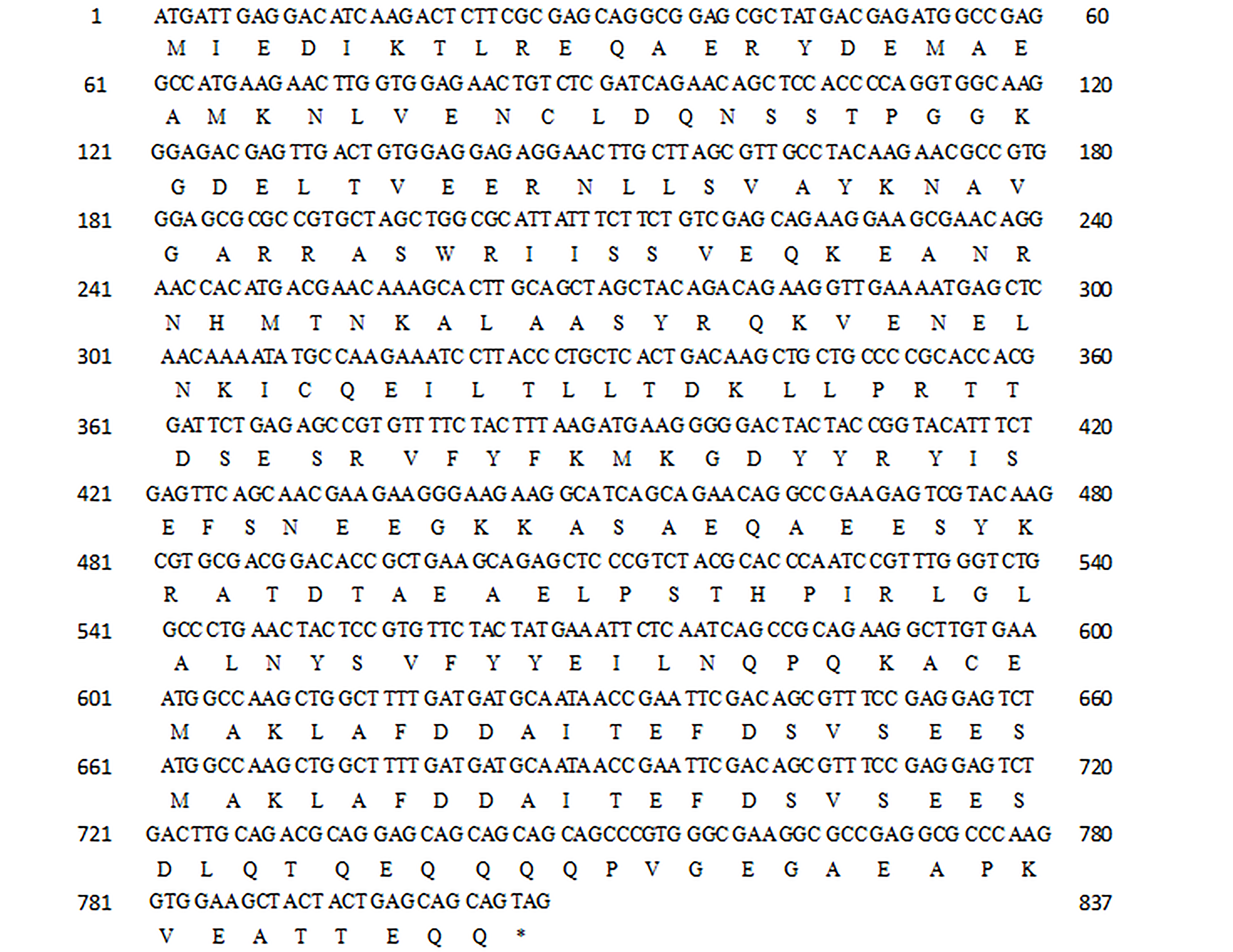

Supplement: Supplementary file 1 — Figure S1. Open reading frames (ORFs) and deduced amino acid sequence of common antigen 14–3-3. (TIF 12897 kb) [file 12917_2018_1665_MOESM1_ESM.tif]

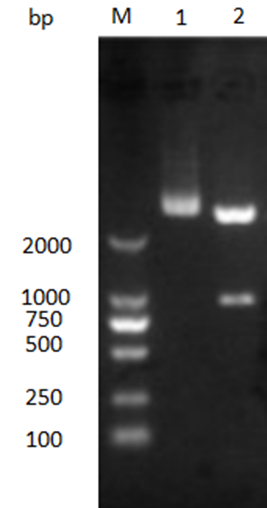

Supplement: Supplementary file 3 — Figure S2. Identification of recombinant plasmid pVAX-Ea14–3-3 digested by BamH I/Xho I. M: DNA molecular weight marker DL 2000. Lane 1: pVAX-Ea14–3-3. Lane 2: pVAX-Ea14–3-3 digested by BamH I/Xho I. (TIF 62 kb) [file 12917_2018_1665_MOESM3_ESM.tif]
