# Supplementary material for: Protective immunity induced by Eimeria common antigen 14–3-3 against Eimeria tenella, Eimeria acervulina and Eimeria maxima
Source: BMC Vet Res. 2018 Nov 12;14:337. doi: 10.1186/s12917-018-1665-z (PMC6233286; doi:10.1186/s12917-018-1665-z)
Supplement: Supplementary file 2 — Table S1. Amino acid similarities of 14–3-3 between E. acervulina, E. maxima, E. tenella, E. necatrix (%). 1.Ea14–3-3 = 14–3-3 of E. acervulina; Em14–3-3 = 14–3-3 of E. maxima; Et14–3-3 = 14–3-3 of E. tenella; En14–3-3 = 14–3-3 of E. necatrix. (DOCX 16 kb) [file 12917_2018_1665_MOESM2_ESM.docx]

Table S1 Amino acid similarities of 14-3-3 between *E. acervulina*, *E. maxima*, *E. tenella*, *E. necatrix* (%)

|  | Ea14-3-3 | Em14-3-3 | Et14-3-3 | En14-3-3 |
| --- | --- | --- | --- | --- |
| Ea14-3-3 | \ | 96.5 | 97.5 | 97.5 |
| Em14-3-3 | 96.5 | \ | 94.1 | 94.1 |
| Et14-3-3 | 97.5 | 94.1 | \ | 100 |
| En14-3-3 | 97.5 | 94.1 | 100 | \ |

Note: 1.Ea14-3-3=14-3-3 of *E. acervulina*; Em14-3-3=14-3-3 of *E. maxima*; Et14-3-3= 14-3-3 of *E. tenella*; En14-3-3=14-3-3 of *E. necatrix.*
